# Flotation of Smithsonite From Quartz Using Pyrophyllite Nanoparticles as the Natural Non-toxic Collector

**DOI:** 10.3389/fchem.2021.743482

**Published:** 2021-10-15

**Authors:** Gaochan Pan, Dan Zou, Zhen Wang

**Affiliations:** ^1^ School of Minerals Processing and Bio-engineering, Central South University, Changsha, China; ^2^ Hunan Research Institute for Nonferrous Metals Co., Ltd, Changsha, China; ^3^ School of Environment and Resource, Southwest University of Science and Technology, Mianyang, China

**Keywords:** nanoparticles, natural hydrophobic, smithsonite, surface potential, adsorption

## Abstract

The use of natural hydrophobic mineral nanoparticles as a collector in froth flotation has recently attracted the attention of researchers. In this article, the separation performance and mechanism of pyrophyllite nanoparticles (PNPs) on smithsonite and quartz flotation system were investigated using the method of flotation, zeta potential, contact angle, and scanning electron microscope (SEM)/energy disperse spectroscopy (EDS). The results of single mineral flotation showed that the difference in flotation recovery between smithsonite and quartz was large for NaOL, DDA, and PNP collectors in the acidic pH range, the largest of which was the PNP system. At pH 6, the optimal dosage of PNPs was 1,000 mg/L. Separation of mixed minerals of smithsonite and quartz using a PNP collector provides the optimum concentrate index (Zn grade 50.84% and Zn recovery 85.36%). According to the results of zeta potential measurement, PNPs and quartz were negatively charged, and the surface of smithsonite was positively charged at pH 6. This provided conditions for smithsonite to selectively adsorb PNPs due to different electrostatic forces. Selective adsorption of PNPs in the smithsonite/quartz flotation system was directly observed by SEM/EDS detection. Hydrophobic PNPs were adsorbed on the surface of hydrophilic smithsonite to make it hydrophobic, and the surface of quartz remained hydrophilic. This is the mechanism for separating smithsonite and quartz using PNPs.

## Introduction

The collector is the most important reagent used in ore flotation, and the pH regulator, activator, depressant, and frother are all used to create conditions for the collector to play a better role (de Medeiros and Baltar, 2018). Traditional organic collectors direct their hydrophobic chains to the solution, and the hydrophilic head is adsorbed to the active site on the mineral surface, making the mineral surface hydrophobic (Fuerstenau and Jia, 2004). Hydrophobic mineral particles can collide with bubbles and attach to them, lifting them onto the surface of the pulp (Marion et al., 2020). Therefore, much research has focused on the development of highly efficient collectors of various minerals. Oleates, amines, xanthates, sulfonates, etc. and mixtures thereof are the most commonly used collectors in the flotation process (Vidyadhar and Rao, 2007; Ejtemaei et al., 2014; Shu et al., 2020). There is evidence to suggest that the use of these reagents causes many environmental problems and threatens human health due to their degradation resistance or high toxicity (Chen et al., 2011; Fu et al., 2020). The research and development of environmentally friendly collectors in the flotation industry is related to the sustainable development of technology.

Silicate minerals are common gangues associated with metal ores, and some literature studies report them as collectors (Henne et al., 2018; Hajati et al., 2019). All types and contents of silicate minerals are abundant in the Earth’s crust and even moon rocks (Huebner and Dillenburg, 1995). According to the connecting format of [SiO_4_] tetrahedron in the molecular structure, the silicate minerals are divided into nesosilicate, chain silicate, ring silicate, tectosilicate, and phyllosilicate (Ramberg, 1952). Among these, phyllosilicate, the so-called tetrahedral–octahedral–tetrahedral (TOT) type, is the most studied. Talc, chlorite, mica, pyrophyllite, etc. are the well-studied phyllosilicate minerals in different fields. Because the Al atoms in pyrophyllite exist within the structure of the octahedral sheet, they do not appear on cleavage surfaces during grinding in mill (Yariv and Cross, 1979). Cleavage occurs between two tetrahedral sheets in adjacent layers, resulting in lower free energy on the exposed surface of the ground pyrophyllite particles and increased hydrophobicity of the particles (Newton and Sposito, 2015).

Given the demand for green collectors and the abundant hydrophobic pyrophyllite resources, it is easy to raise the question of whether pyrophyllite particles can be used as collectors. In fact, research into the use of natural mineral nanoparticles such as flotation reagents has been conducted in recent years. Serpentinite, a common natural mineral generated in sulfide ore, has been found to be a potential selective inhibitor of pyrite for flotation galena from pyrite (Feng et al., 2019). In this report, -10 μm serpentinite was used, but it was found that the serpentinite nanoparticles were the actual functional components. Natural hydrophobic talc nanoparticles have been implemented as collectors for quartz flotation, and with this new collector system, the optimum particle size for quartz was also about −125 + 38 μm, similar to a typical collector system (Hajati, et al., 2016). And the smaller the size of the talc nanoparticle collector was, the stronger the collecting power would be. It was also used for flotation of malachite, a refractory metal oxide mineral (Choi, et al., 2020). The pH range between the IEP of natural mineral nanoparticles and the target mineral is important for an inhibitor or collector. In this range, the nanoparticles and target minerals are oppositely charged, allowing electrostatic attraction and then depression/collection.

To clarify the feasibility of natural mineral nanoparticle collectors in flotation, researchers continue to study a variety of mineral nanoparticle species. Although collector development must ultimately achieve mineral separation, almost all reported results are from a single mineral system (except for mineral nanoparticle collectors). Here, smithsonite, a representative refractory metal oxide mineral, and its common associated gangue mineral quartz were employed as the study object, and pyrophyllite nanoparticles, sodium oleate (NaOL), or dodecylamine (DDA) was used as the collector. Pyrophyllite is another natural hydrophobic mineral similar to talc. The separation effect and mechanism of smithsonite and quartz were discussed using this new mineral nanoparticle collector.

## Materials and Methods

### Mineral Samples and Chemical Reagents

**FIGURE 1:**
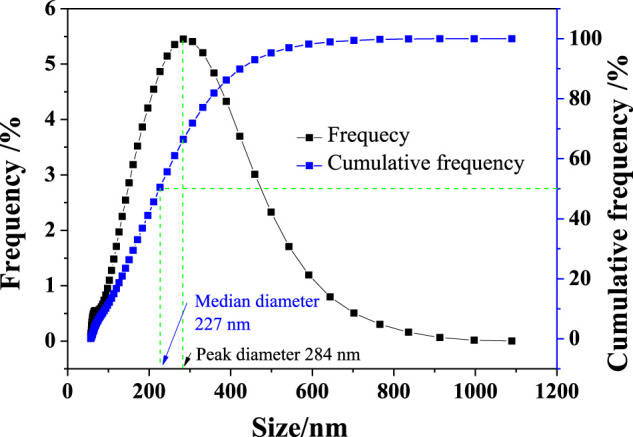
Size distribution curve of PNP.

**FIGURE 2:**
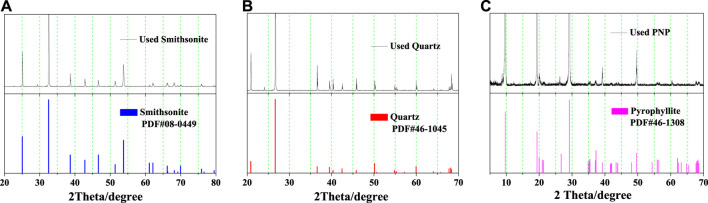
XRD graphics of **(A)** smithsonite, **(B)** quartz, and **(C)** PNP.

Pure samples of smithsonite and quartz minerals were obtained from Huili, Sichuan Province, China. The two samples were manually crushed using a ceramic hammer, hand-sorted, finely ground in a ceramic ball, and dry-screened to collect the −74 + 37-μm and −37-μm fractions of each mineral. Pyrophyllite nanoparticles (PNPs) were obtained from Jiangxi Guangyuan Chemical Industry Co. Ltd, China. The particle size distribution of PNPs is shown in Figure 1. The median diameter of the PNP sample was 227 nm, and the peak diameter was 284 nm. Chemical analysis showed that the three samples had very high purity (>95%), and this was demonstrated by the comparison between their XRD spectra and corresponding PDF card extracted from Jade 6.0 package (Figure 2). The XRD spectra of the pure minerals used in this study had very few impure peaks.

NaOL and DDA were used as the collector in the control groups. Methyl isobutyl carbinol (MIBC) was used as the frother. Hydrochloric acid (HCl) and sodium hydroxide (NaOH) were used for pH control. These reagents were all obtained from the online store Sinopharm Group Co., Ltd. Deionized distilled water (18.3 MΩ cm) was used for all the tests.

### Flotation Tests

Flotation tests were conducted in a mechanical agitation flotation machine (XFG^II^ manufactured by Jinlin Exploration Mechinery Plant, China) equipped with a 40-ml micro-flotation cell. The amount of smithsonite or quartz for each single mineral test was 2.0 g, while for the mixed minerals test, 1.0 g of smithsonite and 1.0 g of quartz were mixed together as the feed sample. After rinsing three times with distilled water, the samples were suspended in the micro-flotation cell with 40 ml distilled water. After stirring and conditioning for 2 min, the desired pH value was adjusted and maintained by HCl or NaOH for another 2 min. Then, the collector and frother were added successively with 3- and 2-min condition time, respectively. Flotation froth was then collected for 5 min. The froth and non-floated particles were collected, filtered, and dried. The volumetric titration method was used for Zn grade determination. Each test at the same condition was performed three times, and the average value was reported.

### Zeta Potential Measurements

The −37 μm fraction of smithsonite and quartz were finely ground to −5 μm, respectively. The zeta potential values were measured at 20 ± 0.5°C using an electrokinetic potential analyzer (ZetaPlus, Bruker, Germany). At a given pH, a solution containing 0.02 g of mineral particles and KCl (40 ml, 1 mM) background electrolyte was prepared in a beaker. After allowing the solution to stand for 5 min, the supernatants were taken to measure the zeta potential. It was also performed three times for each measurement at the same condition, and the average value was reported.

### Contact Angle Measurements

Bulk samples of pyrophyllite, smithsonite, and quartz were cut to a 3-cm × 3-cm tablet, respectively. Then one of the surfaces was ground and polished using an automatic target surface processor (Leika EM TXP). The three cleaned samples were immersed in distilled water, and the natural contact angle of the samples was measured by the sessile drop technique (Hong et al., 2017). To measure the contact angle when the smithsonite and quartz surfaces were adjusted with PNPs, smithsonite and quartz tablets were dipped in suspensions containing different concentrations of PNPs and stirred for 30 min each. The contact angle value was determined by recording an image of the prepared sample with water droplets. Three tests were run under the same conditions, and averages were reported.

### Scanning Electron Microscopy

2.0 g smithsonite or quartz particles were placed in plexiglass cell for 2-min conditioning; the pulp pH was adjusted to 6.0 for 2 min, then the PNP was added into the pulp and conditioned for 3 min. These particles were filtered and vacuum-dried at room temperature and then used for tests. Microtopographies and local energy-dispersive spectra (EDS) of the samples were determined using SEM (Zeiss-Sigma 300, Zeiss, Berlin, German) equipped with an EDS detector (EDAX Inc (Washington, DC, USA). The main operating parameters were 10 kV EHT (acceleration voltage), 8.1 mm WD (working distance), and 5.0 KX Mag (magnification times).

## Results and Discussion

### Flotation Results

**FIGURE 3:**
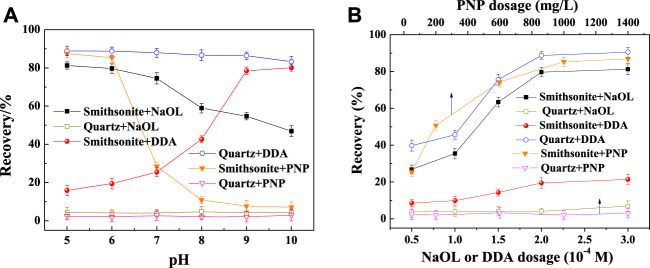
Effect of pulp pH on the floatability of minerals in collector solution [**(A)**, NaOL and DDA 2 × 10^–4^ M, PNP 1 g/L]; and the effect of collector dosage on the floatability of minerals [**(B)**, pH 6.0].

Pulp pH has a significant effect on the flotation of minerals, so the optimal pH for different collectors was first determined. The results are shown in Figure 3. With 2 × 10^–4^ M NaOL as the collector, smithsonite floated well at acid pH (>70% recovery), while the recovery of quartz was at a low level (<10%). The results were similar to those shown in the relevant article (Liu et al., 2019). This showed that smithsonite and quartz were easy to separate with NaOL in the acid pH range. In DDA solution, quartz had good floatability, while smithsonite floated poorly when the pH was less than 8, which suggested that the two minerals might be separated by reversed flotation. However, in actual ore system, smithsonite cannot be the non-floated fraction. When the PNP was used as the collector, quartz showed the lowest recovery in the whole pH range, while smithsonite floated well when the pH was less than 6. If the pH higher than 7, both minerals cannot be floated by PNPs. It showed that at pH 5–6 smithsonite might be separated well from quartz with a PNP collector.

The effect of collector dosage on the recovery of minerals was also investigated. Generally, the floatability of mineral becomes better with the increasing collector dosage until a certain critical value. This was similar to common organic collectors (Vieira and Peres, 2007). These critical values were 1 g/L for PNPs and 2 × 10^–4^ M for NaOL and DDA.

**FIGURE 4:**
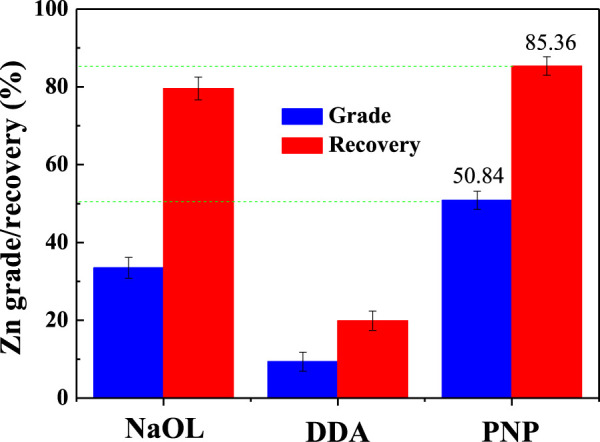
Flotation index of the froth with different collectors (pH 6.0, NaOL, and DDA 2 × 10^–4^ M, PNP 1 g/L).

From the results of a single mineral flotation, it seemed difficult to determine the optimal collector for the smithsonite/quartz separation system. Therefore, the mixed ore system was also examined, and the Zn grade/recovery of the froth product was calculated. The results are shown in Figure 4. Since the DDA could only achieve smithsonite reverse flotation, it showed the worst flotation index and the reverse flotation process were not practical for zinc ore flotation. For NaOL collectors, it was not good when viewed with a single mineral system. The recovery rate of Zn was high, but the grade was relatively low. This may be due to the activation of quartz flotation by the dissolved Zn ions from the smithsonite surface (Ejtemaei et al., 2012). For PNP collectors, the optimal concentration Zn grade was 50.84% and the Zn recovery was 85.36%.

The adsorption mechanisms of NaOL and DDA with smithsonite and quartz have been studied in detail in previous publications. Therefore, the following will focus on the flotation separation mechanism of PNPs in the smithsonite/quartz system.

### Zeta Potentials

**FIGURE 5:**
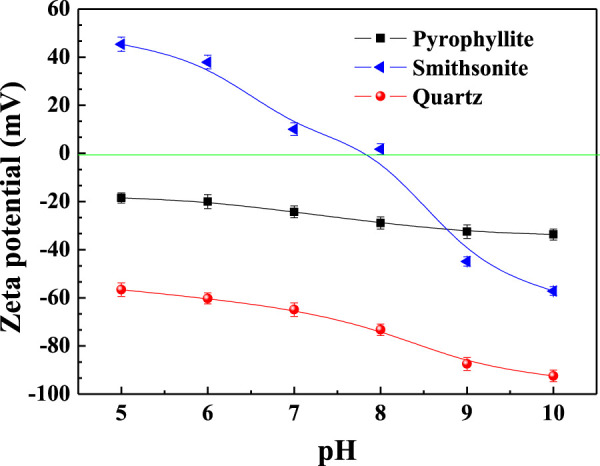
Effect of pH on the zeta potential of pyrophyllite, smithsonite, and quartz particles.

The electrokinetic behaviors of pyrophyllite, smithsonite, and quartz particles as a function of pH are shown in Figure 5. In the tested pH range, the isoelectric points (IEPs) of pyrophyllite and quartz were not found. The IEP of smithsonite was easy to find at about 7.9 in Figure 5, which was very close to the reported values of 8.0 (Hosseini and Forssberg, 2006) and 7.8 (Souza and Lima, 2019). The distinct difference in zeta potential behavior of these three minerals made it possible for them to interact with each other through electrostatic attraction.

At pH 6, the smithsonite surface was positively charged with a zeta potential of 37.93 mV, while the quartz surface was negatively charged with a zeta potential of −60.24 mV. This indicated that smithsonite could be adsorbed by a negatively charged matter, such as PNP and NaOL anions, while quartz could not, which was the reason for the larger floatability difference between the two minerals, with NaOL or PNP as the collector. The mechanism was similar for reverse flotation by the DDA. The aforementioned process still maintains pH 7, but if the zeta potential of smithsonite drops significantly, the electrostatic attraction may not be strong enough. The lower acidic pH 5 may result in serious corrosion in steel equipment and major smithsonite (carbonate mineral) dissolution; this is economically unviable in the industrial process. So, pH 6 should be regarded as the optimal pH for the separation of smithsonite and quartz with a PNP collector.

### Contact Angles

**FIGURE 6:**
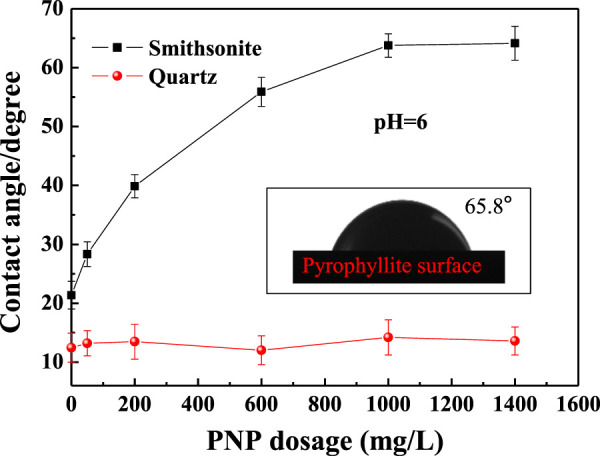
Contact angles for smithsonite and quartz after treatment with a different dosage of PNP.

To clarify the understanding of the results regarding the flotation behavior of smithsonite and quartz with different concentrations of PNPs, the influence of the PNP dosage on the hydrophobicity of smithsonite and quartz surface was investigated through contact angle measurement. The results are shown in Figure 6. The bare pyrophyllite surface had a contact angle of 65.8° (inset), displaying certain natural hydrophobicity (Hu et al., 2003), while bare smithsonite and quartz had contact angles of 21.37° and 12.45°, respectively, showing natural hydrophilicity.

Increasing the PNP dosage to 1,000 mg/L clearly increased the contact angle on the smithsonite surface to 63.77°. When the PNP dosage range is greater, this dependency was not noticeable. For quartz, PNP treatment does not significantly affect the surface contact angle. It maintained a little more than 10°, close to the value on a bare quartz surface. Therefore, at pH 6, the hydrophobic PNP was adsorbed on the smithsonite surface, and the electrostatic attraction between the negatively charged PNP and the positively charged smithsonite particles produced a hydrophobic smithsonite surface, thus promoting smithsonite flotation. On the other hand, the PNP could not be adsorbed on negatively charged quartz particles. This means that the different surface charge of smithsonite and quartz at pH 6 resulted in the different adsorption behaviors of PNPs, causing different hydrophobic properties of the two surfaces, and ultimately caused different flotation behaviors of smithsonite and quartz.

### Scanning Electron Microscope Observations and Energy Disperse Spectroscopy Detections

**FIGURE 7:**
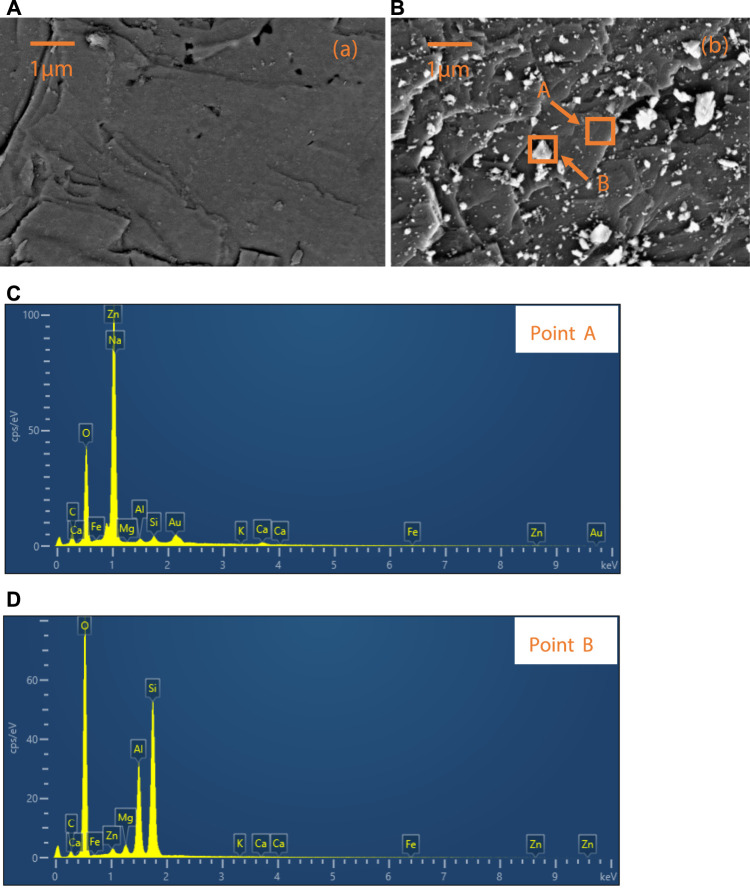
**(A)** Quartz and **(B)** smithsonite surface after treatment with PNP, and the EDS results of **(C)** point A and **(D)** point B on the treated smithsonite surface.

According to the results of the zeta potential test, the electrostatic attraction between PNPs and smithsonite particles and the electrostatic repulsion between PNPs and quartz particles may cause PNPs to be selectively adsorbed on smithsonite. Here, SEM/EDS analysis was employed to confirm whether this phenomenon has occurred. The results are shown in Figure 7.

Figure 7A shows the quartz surface after PNP treatment; except some nicks, no obvious granular substance was found, while on the PNP-treated smithsonite surface, many particles could be found, as shown in Figure 7B. The size of these small granular materials ranges from tens to hundreds of nanometers, which is consistent with the results of the PNP size distribution (see Figure 1).

Two points on the smithsonite surface were selected to conduct the EDS detection, and they were signed as point A and point B. Point A was the clean position of the smithsonite surface with adsorbed PNPs; point B was one of the granular materials adsorbed on the smithsonite surface. The results are displayed in Figures 7C,D, respectively. The main elements of point A were Zn, O, and C, according to the element constituent of smithsonite (ZnCO_3_). Au element that occurred on the surface may be ascribed to the gold plating process, by which surface topography of high quality could be obtained. Other elements should come from the impurities present in the smithsonite. When point B was detected, it can be found that the main elements of the adsorbed particulate matters are Si, Al, and O, which is consistent with the element of pyrophyllite (Al_2_[Si_4_O_10_](OH)_2_) (Evans and Guggenheim, 1988). So, now it was confirmed that the PNPs were selectively adsorbed onto smithsonite surface compared with quartz.

### Interaction Mechanism Analysis

So far, the adoption of natural hydrophobic mineral nanoparticles as flotation collectors is due to the electrostatic interaction (physical adsorption) between the floating minerals and NP. (Legawiec and Polowczyk, 2020). Therefore, controlling the pH of pulp is important based on its large effect on the surface charge of minerals. The NP modification process may allow chemical adsorption between the NP and the mineral of interest to enhance the interaction, improve flotation, and even break through pH range limits.

**FIGURE 8:**
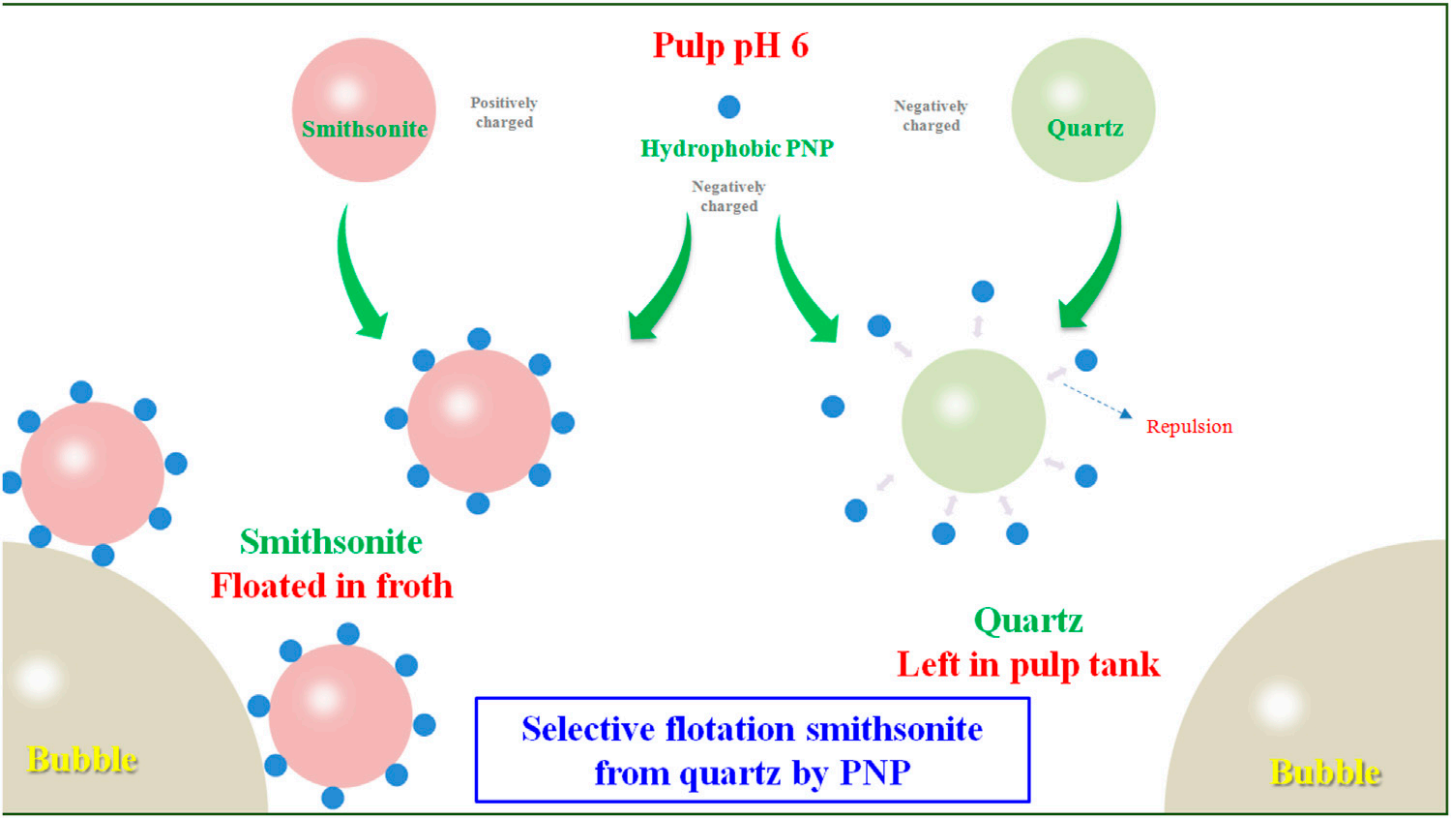
Model schematic diagram for PNP flotation separation smithsonite from quartz.

A model for PNP selective flotation hydrophilic smithsonite from hydrophilic quartz at pH 6 was proposed and is displayed in Figure 8. The pH 6 of pulp was higher than that of the IEP of quartz (∼2.0), and pyrophyllite (∼2.7), so they were negatively charged; but it was lower than the smithsonite IEP 7.9, so the surface of smithsonite was positively charged (Figure 5). When the hydrophobic PNP was added to the smithsonite pulp, it was adsorbed onto the smithsonite surface by electrostatic attraction and made it hydrophobic. The hydrophobic smithsonite particles then collide and adhere to the gas bubble and then were floated with froth fraction, forming the smithsonite concentrate. When negatively charged, the PNP was added in negatively charged quartz particles pulp, it was not adsorbed because there was electrostatic repulsion between them. The still hydrophilic quartz particles then collided but did not adhere on the gas bubble, and then were left in the pulp tank forming tailings. For the mixed minerals system the electrostatic attraction between smithsonite and quartz particles was small due to their big size and thus, large interaction distance. Due to vigorous agitation in the pulp tank, the electrostatic attraction between smithsonite and quartz broke more easily than the electrostatic attraction between PNPs and smithsonite. Therefore, in mixed minerals, the pulp PNP was found to still have excellent performance in separating smithsonite and quartz (Figure 4).

## Conclusion

In this study, nanoparticles of a natural hydrophobic mineral, pyrophyllite nanoparticles (PNPs), were investigated as collectors of smithsonite and quartz flotation systems. The difference in floatability between smithsonite and quartz reached its maximum at pH 6 with PNPs as the collector. PNPs showed good selectivity for the smithsonite/quartz flotation system. For mixed minerals, zinc concentrates with a zinc grade of 50.84% and a zinc recovery of 85.36% can be obtained using PNPs as a collector at pH 6. The flotation performance of PNPs is better than that of the NaOL or DDA collector for this mineral system. At pH 6, PNP and quartz are negatively charged and the surface of smithsonite is positively charged, so the PNP was adsorbed on the surface of smithsonite but not on the surface of quartz. This was confirmed by the SEM/EDS results. The contact angle of pyrophyllite, smithsonite, and quartz are 65.8°, 21.37°, and 12.45°, respectively. The adsorbed hydrophobic PNP made the smithsonite surface hydrophobic, but the quartz surface remained hydrophilic. This is the mechanism by which the PNP is used to separate smithsonite and quartz. Natural hydrophobic pyrophyllite can serve as a non-toxic collector for flotation of smithsonite from quartz.

## Data Availability

The data presented in the study are deposited in the figshare repository, accession number (10.6084/m9.figshare.16704937).
